# PreImplantation factor (PIF) potentiates static magnetic field (SMF) effect to decrease tumor burden (melanoma murine model)

**DOI:** 10.1186/2051-1426-1-S1-P80

**Published:** 2013-11-07

**Authors:** Beatrix Kotlan, Janos F Laszlo, Jozsef Tovari, Miklos Kasler, Eytan Barnea

**Affiliations:** 1 Molecular Immunology and Toxicology, National Institute of Oncology, Budapest, Hungary; 2 Surgical and Molecular Tumorpathology, National Institute of Oncology, Budapest, Hungary; 3 Applied Mathematics and Probability Theory, University of Debrecen, Faculty of Informatics, Debrecen, Hungary; 4 Experimental Pharmacology, National Institute of Oncology, Budapest, Hungary; 5 Board of Directors, National Institute of Oncology, Budapest, Hungary; 6 Univ Medicine and Pharmacy, Tirgu Mures, Romania; 7 Society for the Investigation of Early Pregnancy, Cherry Hill, NJ, USA; 8 BioIncept LLC, Cherry Hill, NJ, USA

## Background and objectives

PreImplantation factor (PIF) secreted by viable embryos exerts essential regulatory role on global systemic immune response. Synthetic PIF (PIF) translates endogenous effects to immune disorder models. Metastatic melanoma displays tumor-immunological behaviour. Static magnetic field (SMF) affects inflammatory reactions. There is increased interest toward SMF’s potential anti-tumor effects. Herein examined a novel anti-melanoma strategy using combined physical and immune-based therapy.

## Methods

Daily whole-body SMF exposure, combined with subcutaneous PIF administration was examined in engrafted HT199 melanoma cells’ progression, transplanted into NSG mice. PIF effect on unique tumor-associated antigen expression relevant for tumor proliferation/ invasion was examined in vitro using specific antibodies. Direct PIF anti-proliferative effects on several cancer cell lines were tested using MTT.

## Results

PIF potentiates SMF beneficial effect by reducing tumor volume vs. control (Mmax=96%) on day 34. Metastatic spleen mass is reduced by SMF alone (M=59%) or combined with PIF (M=62%). Daily SMF exposure alone inhibits tumor outgrowth (Mmax=60%, F5.32 (P<0.002)=21.16) while in combination with PIF, effect is considerably potentiated (M=80%), F5.32(P<0.0004)=34.84). PIF did not impair tumor antigen expression nor reduced significantly cultured tumor cell lines’ proliferation.

## Conclusions

Collectively, results indicate that PIF’s potentiating anti-tumoral effect is mainly immune-regulatory, synergizing with SMF’s pro-tumor necrosis properties. The preserved tumor-associated antigen expression is important for the maintained antitumor immune activity. Overall, combined physico / immune regulatory treatment represents a useful, promising novel avenue for anti-cancer strategy.

